# Evaluation of a quantitative method for measuring the spatial resolution of a gamma camera using a bar phantom^☆^

**DOI:** 10.1016/j.zemedi.2026.02.004

**Published:** 2026-02-18

**Authors:** Jule Maack, Christian Schütze

**Affiliations:** Department of Nuclear Medicine, University Medical Center Goettingen, Robert-Koch-Strasse 40, 37075 Goettingen, Germany

**Keywords:** Constancy checks, Quality assurance, Gamma camera, Spatial resolution, Modulation transfer function, *FWHM*

## Abstract

The method developed by Hander et al. (1997) [1] enables a quantitative determination of spatial resolution by analysing planar images of a lead bar phantom. The method is based on the determination of the modulation transfer function (MTF) and the full width at half maximum (FWHM) solely on the basis of the mean value and standard deviation within a region of interest (ROI). To assess the applicability of this method for constancy checks with extrinsic measurements, the influence of the phantom-to-collimator distance, the total counts and the matrix size was evaluated. In addition, the constancy of the method for repeated measurements under repositioning was analysed.

The phantom-to-collimator distance significantly affects the determined resolution, with the *FWHM* increasing by approximately 0.4 mm per centimetre of distance. The matrix size also influences the determined values, leading to up to 20% lower *MTF* values for the larger bars (4 and 5 mm) when decreasing the matrix from a 1024 × 1024 to a 256 × 256 matrix. The total number of counts does not affect the *MTF* or *FWHM* values, provided that at least 3·10^6^ counts are acquired. However, this applies only to the larger bars (4 and 5 mm); if reliable values are required for all four quadrants, the total number of counts should be increased further. Under constant measurement conditions, variations below 2.4% were observed for the bars of 4 and 5 mm width. For the smaller bars of 2 and 3 mm width the variations were below 9%.

## 1 Introduction

Quality Assurance of gamma cameras includes a variety of tests [1], [2]. Spatial resolution is typically tested semi-annually [3] using either a lead bar phantom or an orthogonal hole phantom. However, the evaluation is currently a simple visual inspection of the acquired scintigrams [3], [4]. To enable comparability of the tests and to ensure reproducibility and independence of the evaluating person, Hander et al. [5] developed an evaluation method to obtain the modulation transfer function (*MTF*) and the full width at half maximum (*FWHM*) based on the mean value and the standard deviation in a region of interest (ROI) in the scintigram of a bar phantom. Wasserman [6] transferred these considerations to extrinsic measurements. The aim of this study is to identify potential factors influencing the results when measurement and setup parameters are varied, and to evaluate the applicability of the method in the context of constancy checks.

## 2 Background

To further illustrate the method, the equations applied by Hander et al. [5] are summarised below.

The oscillation of the signal caused by the bars in the phantom can be characterised by the modulation *M*. The modulation transfer function *MTF* relates the input modulation *M_signal_* of the signal at a given frequency *f* to the output modulation *M_output_* of the resulting scintigram. It thereby represents the transfer capacity of the whole system for the modulation at a certain frequency [5], [7]. With a perfect system, the output modulation exactly equals the signal modulation, and the *MTF* equals unity. However, due to the limited resolution, a maximum frequency exists. Beyond this frequency, the output modulation and thereby the *MTF* approaches zero [8].

Hander et al. [5] demonstrated that, in the imaging of a bar phantom, the *MTF* to a given spatial frequency *f* can be determined solely on the basis of the standard deviation σ(f) and the mean value μ(f) within a ROI at this frequency:

(1) $$MTF\left(f\right)=\frac{\sqrt{2}\cdot \sigma \left(f\right)}{\mu (f)}$$

It was further stated that the measurable variance $\sigma_{\mathrm{ROI}}^{2}$ in a given ROI can be divided into three components: in the variance due to the bar pattern ($\sigma_{\mathrm{bars}}^{2}$), due to the statistical noise ($\sigma_{\mathrm{noise}}^{2}$) and due to spatial non-uniformities ($\sigma_{\mathrm{nu}}^{2}$) [5]. The relation between all variances can be defined as [5]:

(2) $$\sigma_{\mathrm{ROI}}^{2}=\sigma_{\mathrm{bars}}^{2}+\sigma_{\mathrm{noise}}^{2}+\sigma_{\mathrm{nu}}^{2}$$

Eq. (1) only takes the standard deviation due to the bars $\sigma_{\mathrm{bars}}$ into account. With a Poisson-distributed noise, the resulting variance $\sigma_{\mathrm{noise}}^{2}$ equals the mean value $\mu_{\mathrm{ROI}}$ in the ROI. The variance due to non-uniformities $\sigma_{\mathrm{nu}}^{2}$ can be considered negligible if the uniformity of the camera and source is sufficient, particularly because, especially for the larger bars, $\sigma_{\mathrm{bars}}^{2}$ dominates [5].

The equation for the *MTF* at a certain bar frequency $f_{b}$ can thereby be rewritten as [5]:

(3) $$MTF\left(f_{b}\right)=\frac{\sqrt{{2(\sigma }_{\mathrm{ROI}}^{2}-\mu_{\mathrm{ROI}})}}{\mu_{\mathrm{ROI}}}$$

Wasserman [6] introduced a prefactor of $\frac{\pi }{4}$ into Eq. (3) for extrinsic measurements. As this constant factor does not affect the constancy of the *MTF* data but reduces the dynamic range of the data due to its absolute value being less than one, the original form as proposed by Hander et al. [5] was used.

When only considering a single gap between two bars, the spatial resolution of the gamma camera can be specified through the full width at half maximum (*FWHM*). To determine the *FWHM*, the line spread function (LSF) is approximated as Gaussian. Since the *MTF* is the Fourier transform of the LSF, the following relation applies, with w denoting the bar width and $\mathrm{MTF}(f_{b})$ representing the *MTF* at the spatial frequency corresponding with the bar width, as determined by Eq. (3) [5]:

(4) $$\mathrm{FWHM}\approx 1.058\cdot w\cdot \sqrt{ln\left(\frac{1}{\mathrm{MTF}(f_{b})}\right)}$$

## 3 Methods

To identify potential factors influencing the values determined with the presented method, measurements from two camera models from different vendors are examined. The measurements are carried out at the Clinic for Nuclear Medicine at Göttingen University Medical Centre using both the Discovery NM 630 (GE Healthcare, USA) and the BrightView (Philips Healthcare, Netherlands).

The measurements are taken extrinsically with a homogeneously filled flood phantom (Pro-project/Pro-NM AutoFlood, Poland) in accordance with DIN 6855-2 [3], using low-energy high-resolution collimators. The lead bar phantom (Pro-Project, Poland) consists of four quadrants with bar widths of 5, 4, 3, and 2 mm. In the flood phantom, ^99m^Tc is used with a mean activity of 450 MBq.

Eqs. (3), (4) are implemented using the open-source platform Fiji ImageJ [9]. According to Hander et al. [5], circular ROIs are used. The ROI diameter is set to 85% of the quadrant height to maximise the analysed area while simultaneously avoiding potential edge effects. With the phantom used in this study, this corresponds to a ROI diameter of 16 cm. Within the individual ROIs, the mean value and standard deviation are determined in order to apply Eq. (3), from which Eq. (4) is subsequently derived.

To account for statistical fluctuations and dependencies, the ROI is moved ten times within each quadrant, and ten individual *MTF* and *FWHM* values are calculated for each quadrant in each measurement. To assess the dependence of the method on the acquisition parameters, the mean of these ten values is used. The standard deviation across the ten measurements serves as an estimate of the statistical measurement uncertainty (see supplement data)

To analyse the dependence of the method on the distance between the bar phantom and the collimator, the distance is gradually increased from 1 cm to 10 cm. Due to the camera design, only distances of 3 cm or more can be realised for the measurements with the Discovery. All measurements are performed with a total number of 8·10^6^ counts. A 512 × 512 matrix is used, as a larger matrix would result in fewer counts per pixel and thus higher noise, while a smaller matrix would yield a pixel size exceeding the smallest bar width.

To investigate the influence of the total number of accumulated counts, measurements with 0.5·10^6^, 3·10^6^, 5.5·10^6^, 8·10^6^, 10·10^6^, 20·10^6^ and 100·10^6^ counts are evaluated. Again, a 512 × 512 matrix is used and the distance is kept as small as possible; with a distance of 1 cm for the measurements with the BrightView and 3 cm for the measurements with the Discovery.

To analyse the effect of the matrix size, sizes of 256 × 256, 512 × 512 and 1024 × 1024 are evaluated. A larger matrix size was not accessible, whereas a smaller matrix size would result in pixel sizes exceeding three of the four examined bar widths. 8·10^6^ counts are accumulated and distances of 2 cm (BrightView) and 3 cm (Discovery) are used.

Finally, the consistency of the method is assessed by analysing six images from each device using consistent imaging parameters. The selected measurement parameters are based on the outcomes of the previous analyses. A 512 × 512 matrix is used, with a distance of 5 cm between the collimator and the bar phantom and a total number of 10·10^6^ counts. Since data consistency, including statistical fluctuations, is investigated, only a single ROI is analysed per measurement and per quadrant. This ROI is positioned at the centre of the respective quadrant, and the resulting value is treated as the only measurement value for that acquisition, analogous to the potential use in constancy testing. Between measurements, the setup was completely disassembled and manually reassembled in the same configuration. Any variation in positioning or orientation arises solely from inevitable human inaccuracies. These small, unintentional differences effectively simulate the variations relevant to routine constancy testing procedures, as no deliberate changes (such as a 90° rotation) were introduced that could add additional influencing factors.

## 4 Results

### 4.1 Phantom-to-collimator distance

**Fig. 1:**
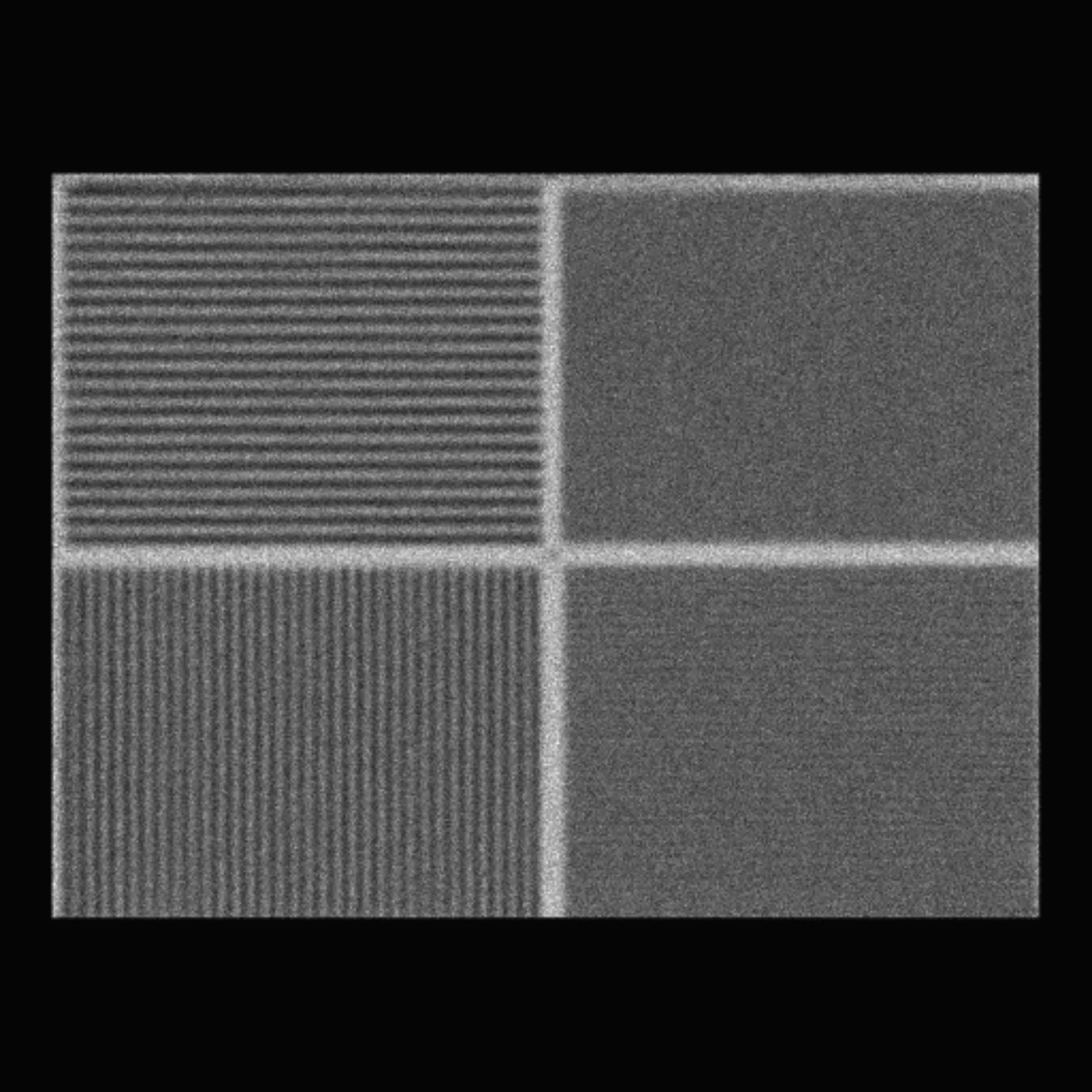
Scintigram of the lead bar phantom; BrightView (Philips Healthcare, Netherlands); distance of 5 cm between phantom and collimator; 512 × 512 matrix; 8·10^6^ counts.

As the distance between the bar phantom and the collimator increases, no recognisable pattern is observed in the scintigram for the 2 mm and 3 mm bars, as shown in Fig. 1. Therefore, only the quadrants with 4 mm and 5 mm wide bars are analysed. To identify analogies with relationships described in literature, the *FWHM* is considered.

**Fig. 2:**
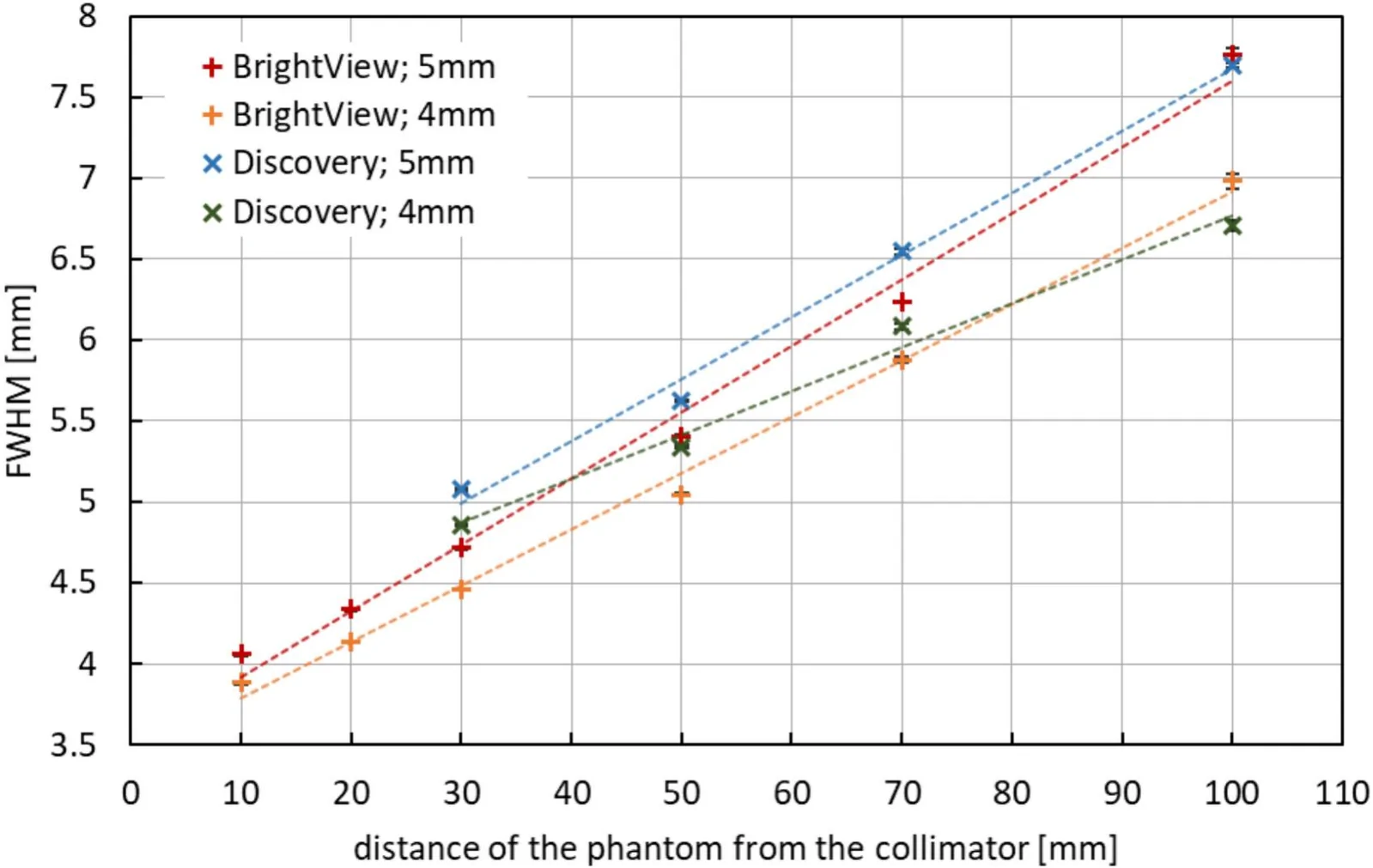
FWHM-values for a lead bar phantom as a function of the distance between the phantom and the collimator for the quadrants with 4 mm and 5 mm wide bars; the data points represent the mean of ten FWHM values obtained from slightly shifted ROIs within the same measurement and quadrant, the error bars indicate the corresponding standard deviation; distinction based on bar width and camera model; linear regression of the data; measurements with Discovery NM 630, GE and BrightView, Philips.

In Fig. 2 the determined *FWHM* are plotted as a function of the distance used. It can be seen that even a small decrease in distance leads to a significant decrease in the *FWHM*. The relationship between the collimator resolution and the distance between the source and the collimator is linear [10], however the *FWHM* presented here illustrates the system resolution. Nevertheless, especially at higher distances, the collimator resolution dominates [11], so the data is examined for a linear relationship. A linear regression of the data leads to an *r^2^* for both cameras and both bar widths of over 0.98. Therefore, the relationship between the *FWHM* determined using this method and the distance between the phantom and the collimator can be seen as linear. For the quadrants with 5 mm wide bars, the *FWHM* increases by 0.4 mm per centimetre of increased distance (BrightView: 0.41 mm/cm; Discovery: 0.38 mm/cm). For the quadrants with 4 mm wide bars, the increase is slightly lower, with 0.3 mm per centimetre of increased distance (BrightView: 0.35 mm/cm; Discovery: 0.27 mm/cm).

Due to the large number of pixels included in the ROI (BrightView: 14,329 pixels; Discovery: 15,984 pixels), the obtained *FWHM* values vary only slightly with repositioning of the ROI. For all distances, both bar widths, and both cameras, a coefficient of variation below 1% is observed.

### 4.2 Total Counts

**Fig. 3:**
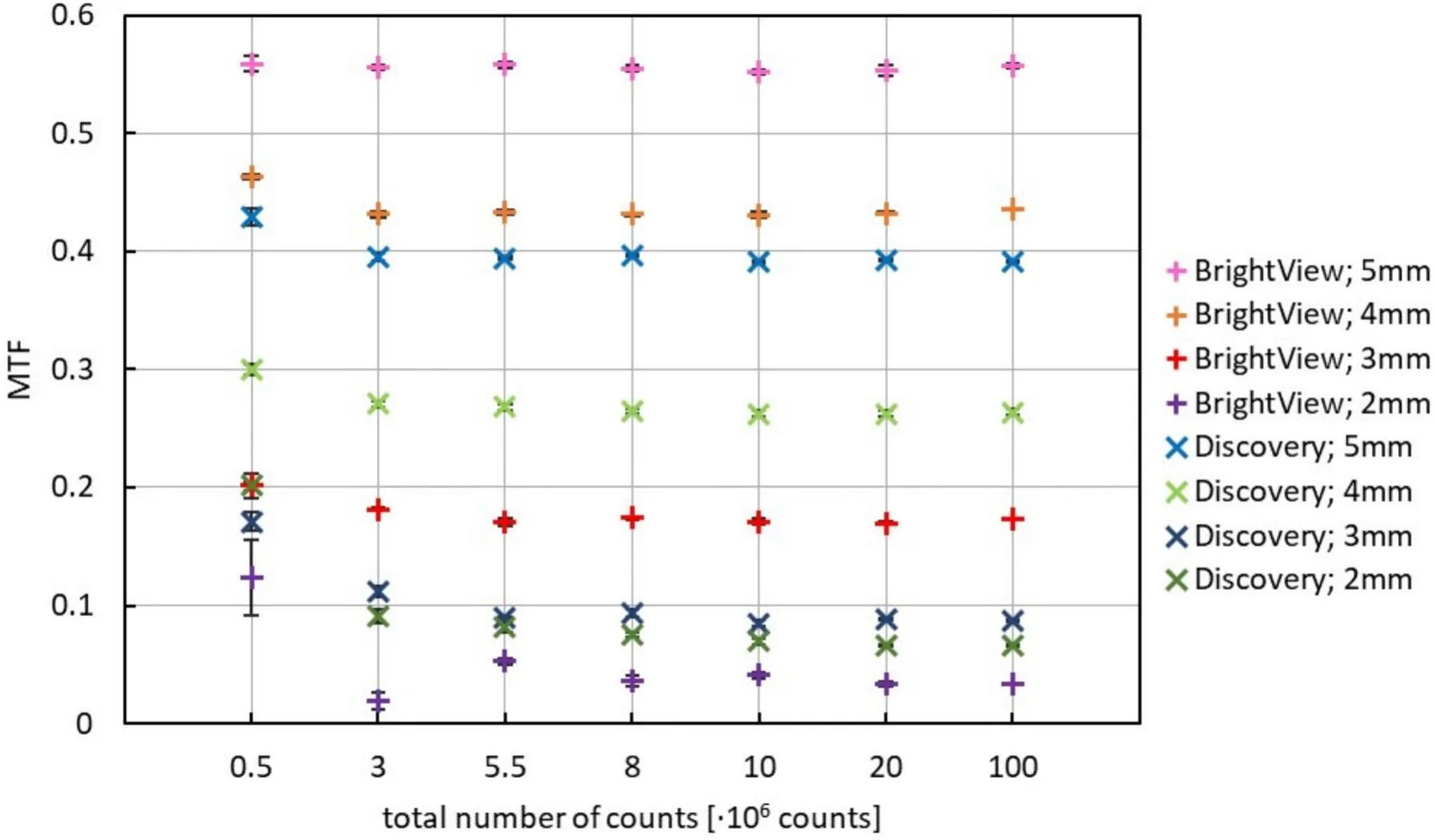
MTF values for a lead bar phantom as a function of the total number of counts, non-scaled count axis; the data points represent the mean of ten MTF values obtained from slightly shifted ROIs within the same measurement and quadrant, the error bars indicate the corresponding standard deviation; distinction based on bar width and camera model; measurements with Discovery NM 630, GE and BrightView, Philips.

The obtained *MTF* values with a variation of the total number of counts are shown in Fig. 3. The *MTF* undergoes only minor fluctuations, despite the varying number of counts. However, this only applies to the measurements with a certain minimum number of counts. With a total number of 0.5·10^6^ counts a higher variation of the *MTF* can be seen. The more counts are obtained, the more stable the *MTF* becomes, even for smaller bars. To quantify and compare the variations, the coefficients of variation of the *MTF* values across measurements with different total counts are given in Table 1, divided by camera model and bar width. The values for the measurement with 0.5·10^6^ counts are not included.

**Table 1:** Coefficients of variation of the MTF when varying the total number of counts; 3·10^6^-100·10^6^ counts; distinction based on bar width and camera model; measurements with Discovery NM 630, GE and BrightView, Philips.

	5 mm	4 mm	3 mm	2 mm
Discovery	0.57%	1.22%	10.79%	13.13%
BrightView	0.42%	0.40%	2.52%	30.14%

The count level also appears to affect the statistical accuracy under repositioning of the ROI within the same measurement. While the *MTF* varies by less than 2% for all bar widths and both cameras in the measurement with 100·10^6^ counts, variations of up to 35.6% are observed at lower count levels. Even for the 4 mm and 5 mm wide bars, variations of up to 1.7% are observed under ROI repositioning when imaging with only 0.5·10^6^ counts.

### 4.3 Matrix

**Fig. 4:**
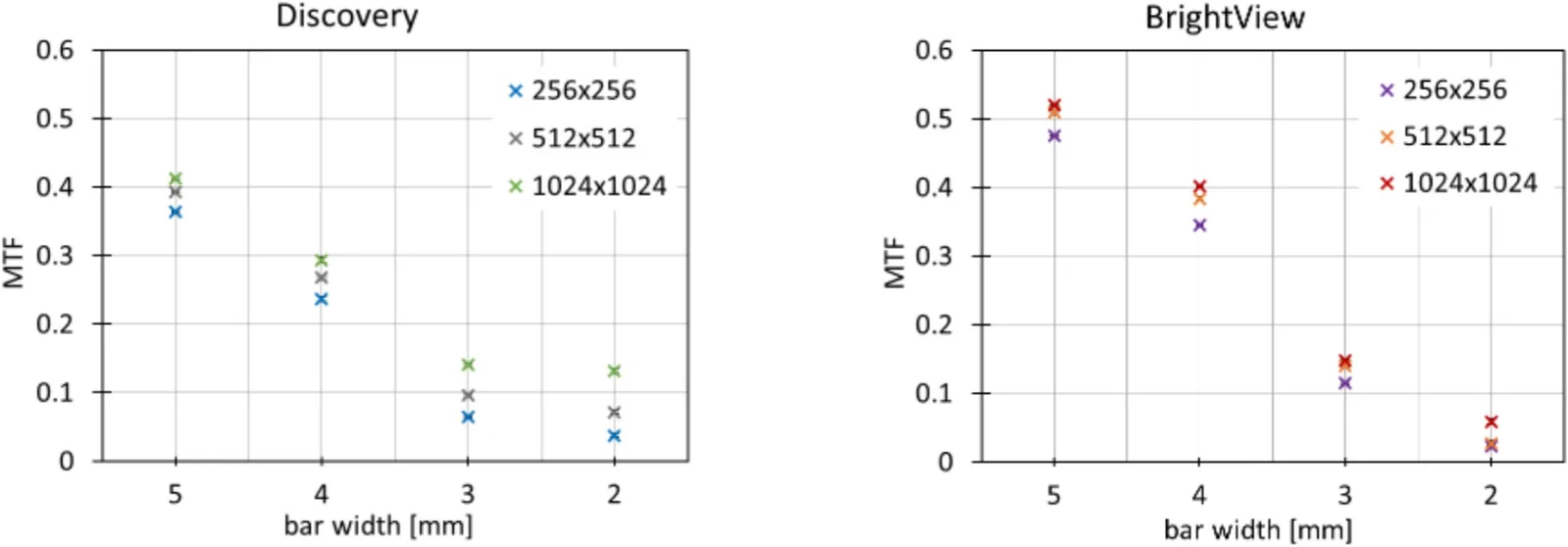
MTF values as a function of the bar width of the lead bar phantom; measurements with Discovery NM 630, GE (left) and BrightView, Philips (right); the data points represent the mean of ten MTF values obtained from slightly shifted ROIs within the same measurement and quadrant, the error bars indicate the corresponding standard deviation; distinction based on the used matrix sizes.

To evaluate the impact of the matrix size, the determined *MTF* values under the presented method for the use of different matrix sizes are shown as a function of bar width in Fig. 4, on the left hand side for the Discovery, on the right hand side for the BrightView. It can be seen that the progression of the *MTF* from quadrant to quadrant is comparable across all matrices for each camera, although the absolute values differ. The larger the matrix, and thus the smaller the pixel size, the higher the *MTF*. For the larger bar widths (4 and 5 mm), the absolute difference between the values obtained with a 1024 × 1024 matrix and a 256 × 256 matrix is approximately 0.05 for both cameras. At this magnitude, this corresponds to a drop in the *MTF* of approximately 20% due to the change in matrix size.

For the smaller bar widths of 2 and 3 mm, the percentage change due to the matrix size is higher. The measurements from the Discovery show larger changes than those from the BrightView. For the 2 mm bars, the absolute difference in the *MTF* for the Discovery is 0.09 when changing from a 256 × 256 to a 1024 × 1024 matrix, resulting in a more than three times higher value for a 1024 × 1024 matrix.

The matrix size does not appear to significantly affect the statistical accuracy under repositioning of the ROI within the same measurement. For the measurements performed here, no matrix size can be identified as exhibiting the highest or lowest variation.

### 4.4 Data consistency

In order to obtain data that is as consistent as possible over a wide range of measurement environments, a distance of 5 cm between the phantom and the collimator is used for the following examinations. Since the 256 × 256 matrix has a pixel size of 2.2 mm × 2.2 mm (Discovery) respectively 2.3 mm × 2.3 mm (BrightView), which is larger than the smallest bar width, a 512 × 512 matrix is used. This enables the highest possible number of counts per pixel without excluding any quadrant. A total number of 10·10^6^ counts is accumulated to minimise the influence of noise.

To quantify the consistency of the data, the coefficients of variation for the *MTF* and *FWHM* are shown in Table 2, divided by bar width and camera model.

**Table 2:** coefficients of variation for the MTF and FWHM values over six repeated measurements per camera with consistent repositioning of the measurement setup; distinction based on used camera model and the bar width; measurements with Discovery NM 630, GE and BrightView, Philips.

	5 mm	4 mm	3 mm	2 mm
Discovery MTF	1.03%	2.34%	3.16%	3.36%
BrightView MTF	0.58%	0.78%	5.26%	8.89%
Discovery FWHM	0.45%	0.74%	0.61%	0.64%
BrightView FWHM	0.27%	0.27%	0.91%	1.35%

In summary, both cameras display an *MTF* variation of less than 2.4% for large bar widths (4 and 5 mm) and less than 9% for small bar widths (2 and 3 mm).

## 5 Discussion

The method presented by Hander et al. [5] is transferable to extrinsic measurements and is easy to apply to the image data.

Due to the large number of pixels within the ROI, the statistical variations under ROI repositioning are small (<2% *MTF*-variation for the 5 mm and 4 mm bars across all count levels, matrix sizes, and distances; with a maximum of 35.6% for the 2 mm bars at 3·10^6^ counts). The observed differences can therefore be regarded as predominantly systematic rather than statistical.

Both Hander et al. [5] and Wasserman [6] classified an *MTF* below 0.1 as tending to be less accurate than higher values, as some limitations of the methodology arise due to the approximations regarding the variances that occur. For the application of the method, good uniformity of both camera and source is necessary, because the variance due to non-uniformities $\sigma_{\mathrm{nu}}^{2}$ is neglected. The extrinsic integral non-uniformity is below 2% for both cameras, and can therefore be considered negligible in the context of the present analysis. Due to the Poisson-distribution of the noise, the resulting variance $\sigma_{\mathrm{noise}}^{2}$ is assumed to be equivalent to the mean value of the ROI [5]. If one of these variances is higher than assumed, the obtained *MTF* according to Eq. (3) will be higher than actually expected. In the framework of constancy checks, however, the absolute accuracy of the determined values is of secondary importance. The focus lies rather on consistency over time and on the method’s responsiveness to deviations in system performance. This can be achieved by constant measurement conditions such as counts, matrix size, and, in particular, phantom-collimator distance, e.g. using the settings applied for the acceptance testing.

It can be shown that for the larger bars (4 and 5 mm bar width) 3·10^6^ counts or more are sufficient to obtain only minor variations due to the changes in the total number of counts. The *MTF*-values for the quadrants with 5 and 4 mm wide bars vary only slightly with less than 1.3%. These values are comparable with the statement by Hander et al. [5], who indicate the influence of the total number of counts with less than 2%. However, it should be noted that they only used a maximum number of 4·10^6^ counts for their intrinsic measurements.

The *MTF* values from the 2 mm quadrant for both cameras, and from the 3 mm quadrant for the measurements with the Discovery, vary with a coefficient of variation exceeding 10%, higher than the remaining values. However, it should be noted that the variation of the values when measuring with the Discovery is only 10.79% for the 3 mm wide bars due to the measurement with 3·10^6^ counts. The bar pattern is hereby hardly visible in the scintigram. However, the *MTF* lies with 0.11 above the average value of the remaining measurements in this series, which is 0.09. Considering only the five measurements with 5.5·10^6^ counts or more, the coefficient of variation for this bar width is 3.58%, comparable to the corresponding value from the BrightView (2.52%).

It is also noticeable that for all quadrants and all bar widths, the *MTF* at 0.5·10^6^ counts is consistently higher than the corresponding values at higher count levels.

This leads to the conclusion that smaller bar widths require more counts for a stable *MTF* determination than larger bar widths. This can be explained by changes in the variations within the scintigram. With a decrease of the total number of counts the approximation regarding the variation due to noise $\sigma_{\mathrm{noise}}^{2}$ tends to be less precise, which explains the higher *MTF* values for the measurement with 0.5·10^6^ counts and the higher variations of the *MTF* for the lower number of counts. For the larger bars, the variance due to the bar pattern $\sigma_{\mathrm{bars}}^{2}$ is larger, so that the influence of inaccuracies in the variance approximations becomes less prominent and the *MTF* values become more stable. This suggests that using a matrix size larger than the 512 × 512 matrix employed here would require an even higher number of counts to achieve stable values.

Apart from the variations associated with the total number of counts shown above, the *MTF* exhibits variations under repeated measurements. According to Hander et al. [5], the variation of the *MTF* for larger bar sizes for repeated intrinsic measurements under the same conditions is lower than 2%. Wasserman [6] reports equivalent extrinsic data, showing a variation below 2.7% when using a high-resolution collimator.

For the measurements with the BrightView at 4 and 5 mm bar widths, as shown in section 4.4, these values can be restricted even further. Here a coefficient of variation of under 1% can be observed. The measurements with the Discovery at 5 mm bar width also confirm the findings by Hander et al. [5], and the quadrant with 4 mm wide bars varies with 2.34% only slightly more. These variations are higher than those observed when varying the total number of counts, but without a consistent repositioning of the measurement setup (<2.4% vs. <1.3%). However, for the smaller bars (2 mm for both cameras, 3 mm for the Discovery), the opposite is observed (<9% vs. <31%).

These effects can also be explained by the approximations made for the variance terms. The measurements according to the data consistency analysis are all obtained with 10·10^6^ counts, resulting in an average of 56 counts per pixel for the Discovery and 60 counts per pixel for the BrightView. The approximation according to the variance due to the noise is therefore sufficient for the larger bars. Here, the influence of the repositioning is higher than the influence of the total number of counts. However, for the smaller bars, not all count levels used appear to be sufficient, so that the variation due to the insufficient number of counts is the dominating variation.

For the application of this method for constancy checks it is thereby necessary to obtain a sufficient number of counts, if reliable and reproducible values are desired for all bar widths.

The distance between the phantom and the collimator has a significant influence on the *FWHM*, with up to 0.4 mm increase per centimetre distance increase. To obtain a comparability between individual measurements, the accuracy of the setup distance is relevant. At the same time, this strong dependence illustrates that the method is sensitive to even moderate degradations in spatial resolution, which underlines its suitability for constancy checks.

The matrix size also has an impact on the determined values, with an up to 20% lower *MTF* for a 256 × 256 matrix compared to a 1024 × 1024 matrix for the larger bar widths. However, to ensure the correct depiction of a spatial frequency, at least two pixels are needed per period to avoid aliasing effects [11]. For the correct depiction of the 2 mm wide bars (with a period of 4 mm), this leads to the need of pixel sizes of 2 mm × 2 mm or smaller. With the camera models used here, the use of a 256 × 256 matrix size leads to a pixel size of 2.2 mm × 2.2 mm (Discovery) respectively 2.3 mm × 2.3 mm (BrightView), so that visualisation of this bar pattern is not possible, regardless of the spatial resolution. A smaller pixel size therefore allows for better representation of the bar pattern and thereby a higher *MTF*, as can be shown by the data here. In addition, the number of counts per pixel decreases as the matrix size increases, so the influence of the variance due to the noise $\sigma_{\mathrm{noise}}^{2}$ becomes greater with larger matrices.

However, this impact is constant for all bar widths. Therefore, all matrix sizes examined here are in principle suitable for the purpose of constancy checks. Nevertheless, it should be kept constant to ensure comparability.

## 6 Conclusions

The method presented by Hander et al. [5] is demonstrated to be applicable for constancy checks, with coefficients of variation below 2.4% for the larger bar widths. However, ensuring the constancy of the measurement setup is crucial to maximise comparability between individual measurements, as well as between devices when comparing results. Especially the distance between the phantom and the collimator should be kept constant, since the *FWHM* increases linearly by 0.4 mm per centimetre of distance. Considering the ease with which the methodology can be implemented and reproduced, and its independence from the individual performing the evaluation, this represents a promising approach for evaluating constancy test recordings. Future studies could examine the influence of the bar phantom in more detail. In addition, the determined values can be examined for correlation with the visual recognisability of the bar pattern.

## CRediT authorship contribution statement

**Jule Maack:** Writing – review & editing, Writing – original draft, Visualization, Methodology, Funding acquisition, Formal analysis, Data curation. **Christian Schütze:** Writing – review & editing, Writing – original draft, Validation, Supervision, Software, Resources, Project administration, Methodology, Investigation, Conceptualization.

## Declaration of competing interest

The authors declare that they have no known competing financial interests or personal relationships that could have appeared to influence the work reported in this paper.

## References

[1] Bundesministerium für Umwelt, Naturschutz und nukleare Sicherheit. Strahlenschutzverordnung (StrlSchV). Verordnung zum Schutz vor der schädlichen Wirkung ionisierender Strahlung. Strahlenschutzverordnung, 29.11.2018 (BGBl. I S. 2034, 2036; 2021 I S. 5261), die zuletzt durch Artikel 1 der Verordnung vom 08.10.2021 (BGBl. I S. 4645) geändert worden ist.

[2] Bundesministerium für Umwelt, Naturschutz, Bau und Reaktorsicherheit. Strahlenschutz in der Medizin - Richtlinie zur Strahlenschutzverordnung (StrlSchV), 26.05.2011 (GMBl. 2011, Nr. 44-47, S. 867). Zuletzt geändert durch RdSchr. des BMUB vom 11.07.2014 (GMBl. 2014, Nr. 49, S. 1020).

[3] Deutsches Institut für Normung. Konstanzprüfung nuklearmedizinischer Messsysteme - Teil 2: Einkristall-Gamma-Kameras zur planaren Szintigraphie und zur Einzel-Photonen-Emissions-Tomographie mit Hilfe rotierender Messköpfe (DIN 6855-2:2013-01); 2013.

[4] Zentraler Erfahrungsaustausch der Ärztlichen Stellen. Einheitliches Bewertungssystem (EBS) der Ärztlichen Stellen nach § 128 StrlSchV. Nuklearmedizin. Version 10.00 (10/2023).

[5] Hander T.A., Lancaster J.L., Kopp D.T., Lasher J.C., Blumhardt R., Fox P.T. (1997). Rapid objective measurement of gamma camera resolution using statistical moments. Med Phys.

[6] Wasserman H.J. (1998). Quantification of gamma camera spatial resolution by means of bar phantom images. Nucl Med Commun.

[7] Ehrhardt JC, Oberley LW, Cuevas JM, Warr CP. Imaging ability of collimators in nuclear medicine. U.S. Department of Health, Education and Welfare 1978. Doi: 10.2172/6291636.

[8] Saha G.B. (2006).

[9] Schindelin J., Arganda-Carreras I., Frise E., Kaynig V., Longair M., Pietzsch T. (2012). Fiji: an open-source platform for biological-image analysis. Nat Methods.

[10] Peter J., Schlegel W., Karger C.P., Jäkel O. (2018). Medizinische Physik: Grundlagen - Bildgebung - Therapie - Technik.

[11] Cherry S.R., Sorenson J.A., Phelps M.E. (2012).

