# Supplementary material for: Evaluation of a quantitative method for measuring the spatial resolution of a gamma camera using a bar phantom
Source: Z Med Phys. 2026 Feb 18;36(3):376–80. doi: 10.1016/j.zemedi.2026.02.004 (PMC13589423; doi:10.1016/j.zemedi.2026.02.004)
Supplement: Supplementary file 1 [file mmc1.docx]

Supplement

To estimate the statistical dependencies, the ROI is shifted ten times in each image and in each quadrant, and the MTF and the FWHM are calculated independently. The mean value and the standard deviation derived from these ten values are provided in this supplement, together with the coefficient of variation. The data on distance variation are given in Table 1, those on count variation in Table 2, and those on matrix variation in Table 3.

Table 1: mean FWHM value, standard deviation, and coefficient of variation for ten shifts of the ROI within the same measurement and the same quadrant; grouped by camera and bar width, as well as by the distance between the phantom and the collimator.

|  | distance [mm] | 10 | 20 | 30 | 50 | 70 | 100 |
| --- | --- | --- | --- | --- | --- | --- | --- |
| BrightView; 5 mm | mean FWHM | 4.058 | 4.338 | 4.716 | 5.407 | 6.237 | 7.760 |
|  | standard deviation | 0.011 | 0.010 | 0.010 | 0.012 | 0.008 | 0.043 |
|  | coefficient of variation | 0.3% | 0.2% | 0.2% | 0.2% | 0.1% | 0.6% |
| BrightView; 4 mm | mean FWHM | 3.882 | 4.135 | 4.458 | 5.044 | 5.877 | 6.981 |
|  | standard deviation | 0.006 | 0.008 | 0.005 | 0.008 | 0.018 | 0.043 |
|  | coefficient of variation | 0.2% | 0.2% | 0.1% | 0.2% | 0.3% | 0.6% |
| Discovery; 5 mm | mean FWHM |  |  | 5.075 | 5.626 | 6.546 | 7.698 |
|  | standard deviation |  |  | 0.009 | 0.005 | 0.022 | 0.018 |
|  | coefficient of variation |  |  | 0.2% | 0.1% | 0.3% | 0.2% |
| Discovery; 4 mm | mean FWHM |  |  | 4.860 | 5.339 | 6.084 | 6.709 |
|  | standard deviation |  |  | 0.005 | 0.009 | 0.015 | 0.034 |
|  | coefficient of variation |  |  | 0.1% | 0.2% | 0.2% | 0.5% |

Table 2: mean MTF value, standard deviation, and coefficient of variation for ten shifts of the ROI within the same measurement and the same quadrant; grouped by camera and bar width, as well as by the total number of counts for the measurement.

|  | number of counts [∙10^6^] | 0.5 | 3 | 5.5 | 8 | 10 | 20 | 100 |
| --- | --- | --- | --- | --- | --- | --- | --- | --- |
| BrightView; 5 mm | mean MTF | 0.559 | 0.556 | 0.558 | 0.555 | 0.552 | 0.553 | 0.557 |
|  | standard deviation | 0.006 | 0.002 | 0.003 | 0.002 | 0.002 | 0.004 | 0.002 |
|  | coefficient of variation | 1.1% | 0.4% | 0.5% | 0.4% | 0.4% | 0.7% | 0.4% |
| BrightView; 4 mm | mean MTF | 0.463 | 0.432 | 0.433 | 0.431 | 0.431 | 0.432 | 0.436 |
|  | standard deviation | 0.003 | 0.003 | 0.002 | 0.002 | 0.002 | 0.001 | 0.001 |
|  | coefficient of variation | 0.5% | 0.6% | 0.4% | 0.4% | 0.5% | 0.3% | 0.3% |
| BrightView; 3 mm | mean MTF | 0.201 | 0.182 | 0.170 | 0.174 | 0.171 | 0.170 | 0.174 |
|  | standard deviation | 0.011 | 0.002 | 0.003 | 0.001 | 0.002 | 0.002 | 0.001 |
|  | coefficient of variation | 5.4% | 0.9% | 1.9% | 0.7% | 1.3% | 1.1% | 0.5% |
| BrightView; 2 mm | mean MTF | 0.124 | 0.019 | 0.053 | 0.036 | 0.041 | 0.034 | 0.033 |
|  | standard deviation | 0.032 | 0.007 | 0.003 | 0.005 | 0.002 | 0.002 | 0.001 |
|  | coefficient of variation | 25.8% | 35.6% | 5.2% | 13.1% | 6.1% | 5.7% | 1.7% |
| Discovery; 5 mm | mean MTF | 0.429 | 0.395 | 0.394 | 0.396 | 0.391 | 0.393 | 0.391 |
|  | standard deviation | 0.007 | 0.003 | 0.001 | 0.001 | 0.001 | 0.001 | 0.001 |
|  | coefficient of variation | 1.7% | 0.7% | 0.3% | 0.2% | 0.4% | 0.2% | 0.2% |
| Discovery; 4 mm | mean MTF | 0.300 | 0.271 | 0.268 | 0.265 | 0.262 | 0.263 | 0.264 |
|  | standard deviation | 0.005 | 0.003 | 0.003 | 0.002 | 0.003 | 0.003 | 0.003 |
|  | coefficient of variation | 1.5% | 1.2% | 1.2% | 0.6% | 1.1% | 1.0% | 1.0% |
| Discovery; 3 mm | mean MTF | 0.171 | 0.112 | 0.089 | 0.094 | 0.085 | 0.089 | 0.087 |
|  | standard deviation | 0.008 | 0.004 | 0.001 | 0.003 | 0.003 | 0.001 | 0.001 |
|  | coefficient of variation | 4.5% | 3.8% | 1.6% | 3.1% | 3.6% | 0.8% | 0.6% |
| Discovery; 2 mm | mean MTF | 0.202 | 0.091 | 0.082 | 0.075 | 0.070 | 0.066 | 0.066 |
|  | standard deviation | 0.004 | 0.006 | 0.005 | 0.002 | 0.003 | 0.001 | 0.001 |
|  | coefficient of variation | 2.0% | 6.5% | 5.7% | 2.4% | 4.1% | 0.8% | 0.8% |

Table 3: mean FWHM value, standard deviation, and coefficient of variation for ten shifts of the ROI within the same measurement and the same quadrant; grouped by camera and bar width, as well as by the matrix size used.

|  | matrix size | 256x256 | 512x512 | 1024x1024 |
| --- | --- | --- | --- | --- |
| BrightView; 5 mm | mean MTF | 0.475 | 0.509 | 0.520 |
|  | standard deviation | 0.001 | 0.003 | 0.001 |
|  | coefficient of variation | 0.2% | 0.5% | 0.2% |
| BrightView; 4 mm | mean MTF | 0.345 | 0.384 | 0.402 |
|  | standard deviation | 0.001 | 0.002 | 0.001 |
|  | coefficient of variation | 0.4% | 0.6% | 0.2% |
| BrightView; 3 mm | mean MTF | 0.115 | 0.140 | 0.148 |
|  | standard deviation | 0.002 | 0.002 | 0.002 |
|  | coefficient of variation | 1.4% | 1.5% | 1.0% |
| BrightView; 2 mm | mean MTF | 0.023 | 0.027 | 0.059 |
|  | standard deviation | 0.003 | 0.004 | 0.004 |
|  | coefficient of variation | 11.7% | 15.8% | 7.2% |
| Discovery; 5 mm | mean MTF | 0.363 | 0.393 | 0.413 |
|  | standard deviation | 0.001 | 0.001 | 0.002 |
|  | coefficient of variation | 0.3% | 0.3% | 0.4% |
| Discovery; 4 mm | mean MTF | 0.236 | 0.268 | 0.293 |
|  | standard deviation | 0.003 | 0.003 | 0.004 |
|  | coefficient of variation | 1.4% | 1.0% | 1.3% |
| Discovery; 3 mm | mean MTF | 0.064 | 0.096 | 0.141 |
|  | standard deviation | 0.002 | 0.002 | 0.002 |
|  | coefficient of variation | 2.6% | 2.0% | 1.4% |
| Discovery; 2 mm | mean MTF | 0.037 | 0.071 | 0.131 |
|  | standard deviation | 0.003 | 0.003 | 0.003 |
|  | coefficient of variation | 7.4% | 3.7% | 2.4% |
